# Low-Intensity Magnetic-Field-Directed Lattice Symmetry Transition to Induce the Centered Rectangular Cylinders in Diblock Copolymer/Magnetic Nanoparticle Nanocomposite Films

**DOI:** 10.1021/acsmacrolett.5c00594

**Published:** 2025-12-16

**Authors:** Wen-Hong Li, Wen-Sheng Chiu, Che-Yi Chu, Ying-Xuan Huang, Yeo-Wan Chiang

**Affiliations:** † Department of Chemical Engineering, 34916 National Chung Hsing University, Taichung 402, Taiwan; ‡ Department of Materials and Optoelectronic Science, 197921 National Sun Yat-sen University, Kaohsiung 804, Taiwan

## Abstract

This study demonstrates a strategy to control lattice symmetry in a polystyrene-*block*-poly­(methyl methacrylate) (PS-*b*-PMMA) diblock copolymer hybridized with a small fraction of NH_2_-tethered Fe_3_O_4_ magnetic nanoparticles incorporated within the cylindrical PMMA microdomains. A low-intensity magnetic field (= 350 mT) was applied following large-amplitude oscillatory shear alignment, transforming the shear-aligned hexagonally packed cylinders (HEX) into centered rectangular cylinders (CR) stabilized through microdomain reorientation that relieved chain crowding and stretching of the PS blocks. In contrast, applying the magnetic field to unoriented HEX induced reorganization into lamellae aligned parallel to the field. The lamellar phase represented the thermodynamic equilibrium state, whereas the CR phase was a kinetically stabilized metastable structure governed by the prealigned framework. These findings highlight low-intensity magnetic manipulation as an effective “noncontact tweezer” for tuning lattice symmetry in block copolymers via the interplay of magnetic anisotropy and initial microdomain orientation.

## Graphical abstract

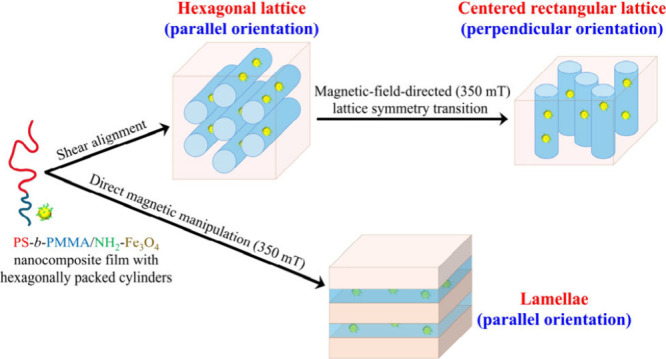

It has been found that block copolymer cylindrical microdomains preferentially adopt a hexagonal lattice in the equilibrium state.^1–4^ Meanwhile, the packing of cylindrical microdomains into a tetragonal lattice^5–11^ or a centered rectangular lattice^12–14^ was also reported. Notably, the latter one with orthorhombic *cmm* symmetry, i.e., the focus of this study, has been extensively characterized in small molecules (such as surfactants,^15–18^ liquid crystals,^19,20^ cationic lipid-DNA complexes,^21,22^ and surfactant-dendrimer complexes^23,24^ ), yet it has rarely been reported in block copolymers.^12–14^ On the other hand, it has been shown that a centered rectangular phase (with lower lattice symmetry) could be induced in liquid crystals by lattice deformation of a hexagonal phase (with higher lattice symmetry), in which temperature played a decisive role in the lattice symmetry transition.^19,20^ Another kinetic pathway for a pressure-induced cubic-to-centered rectangular phase transition was investigated with surfactants.^18^ However, to the best of our knowledge, phase transitions involving the centered rectangular phase in block copolymer systems have not been reported.

In this light, we present the first demonstration of a facilitated block copolymer phase transition from hexagonally packed cylinders (HEX) to centered rectangular cylinders (CR) in an initially HEX-forming polystyrene-*block*-poly­(methyl methacrylate) (PS-*b*-PMMA, further abbreviated as SMMA, where *M*_n,PS_ = 55,000, *M*_n,PMMA_ = 22,000, PDI = 1.08, and *f*_PMMA_ = 25.9 vol %) diblock copolymer hybridized with a low content (= 0.38 vol % with respect to the PMMA phase) of NH_2_-tethered Fe_3_O_4_ (NH_2_–Fe_3_O_4_, with a mean diameter of 6.2 nm for the Fe_3_O_4_ core and a tethering density of 2.9 groups/nm^2^ for the tethered NH_2_ groups on each nanoparticle) magnetic nanoparticles within the cylindrical PMMA microdomains. This phase transition was successfully induced through a combined process comprising shear-induced alignment of the cylindrical microdomains, followed by low-intensity (= 350 mT) magnetic manipulation. Detailed information on the materials and nanocomposite film preparation (as well as morphological characterization by small-angle X-ray scattering (SAXS) and transmission electron microscopy (TEM), and large-amplitude oscillatory shear (LAOS) experiment) can be found in the Supporting Information. In the context of magnetic manipulation of block copolymers, magnetic fields have been shown to accelerate alignment kinetics in liquid-crystalline block copolymers, enabling control of microdomain orientation.^25–28^ By contrast, magnetic-field-directed phase transitions in block copolymer systems remain largely unexplored, despite simulation predictions suggesting their feasibility through the incorporation of magnetic nanoparticles as directing agents.^29^ Our recent studies addressed this gap by demonstrating order–order transitions from HEX to lamellae and to double gyroid in nanocomposites of conventional diblock copolymers and magnetic nanoparticles.^30,31^ Here, we extend this approach to direct lattice symmetry transitions of block copolymer microdomains by using low-intensity magnetic fields.

As identified by SAXS shown in Figure S1 of the Supporting Information, both the neat SMMA diblock copolymer and the SMMA/NH_2_–Fe_3_O_4_ nanocomposite film at 150 °C (well above the glass transition temperatures of the PS and PMMA blocks) exhibited the HEX morphology, as evidenced by a series of scattering peaks showing position ratios of 1:3^1/2^:7^1/2^. This result suggested that the HEX structure was retained after incorporation of NH_2_–Fe_3_O_4_ nanoparticles into the PMMA microdomains via the chemical affinity between the tethered NH_2_ groups and PMMA block chains. It is noted that the suppression of the 4^1/2^ peak for these two samples was consistent with the paracrystalline distortion condition reported for block copolymers whose volume fractions lay near 27.4 vol %, where paracrystalline distortion attenuated specific higher-order reflections such as the 4^1/2^ peak.^32,33^ The volume fractions of the neat SMMA diblock and its blend with NH_2_–Fe_3_O_4_ nanoparticles (= 27.3 and 27.6 vol %, respectively) fell within this range, supporting the notion that the observed extinction originated from paracrystalline distortion of the HEX lattice rather than from compositional asymmetry. This retained HEX structure then served as the initial state for subsequent study.

Importantly, prior to magnetic manipulation, mechanical shear stress was applied to align the NH_2_–Fe_3_O_4_-containing cylindrical PMMA microdomains using LAOS, as illustrated in Figure 1a, with the shear direction (*y* axis) oriented perpendicular to the film surface normal (*z* axis). As further examined by the 2-D SAXS pattern collected from the *y* direction (Figure 1b), the preferred orientation of the hexagonal lattice displayed six arcs with respect to the {10} planes of HEX where six maxima were located by an azimuthal angle interval of 60° (note that the azimuthal scan profile lacked the third and fourth maxima due to the detector’s physical gap, which disrupted the azimuthal integration). Meanwhile, the 2-D SAXS pattern viewed along the *z* direction (Figure 1c) displayed multiple pairs of equatorial arcs arising from periodic stacking of the cylinder axes, confirming the formation of an oriented HEX structure. The observed reflection sequence corresponded to the in-plane projection of the HEX lattice rather than a genuine lamellar morphology. The corresponding 1-D SAXS profiles are shown in Figure 1d to index the peak position ratios of HEX viewed along the *y* and *z* directions. It should be noted that as manifested by the broad higher-order peaks at *q*/*q*_m_ = 3^1/2^, 4^1/2^, 7^1/2^, and 9^1/2^, the long-range order in this shear-aligned nanocomposite film was not high.

**Figure 1:**
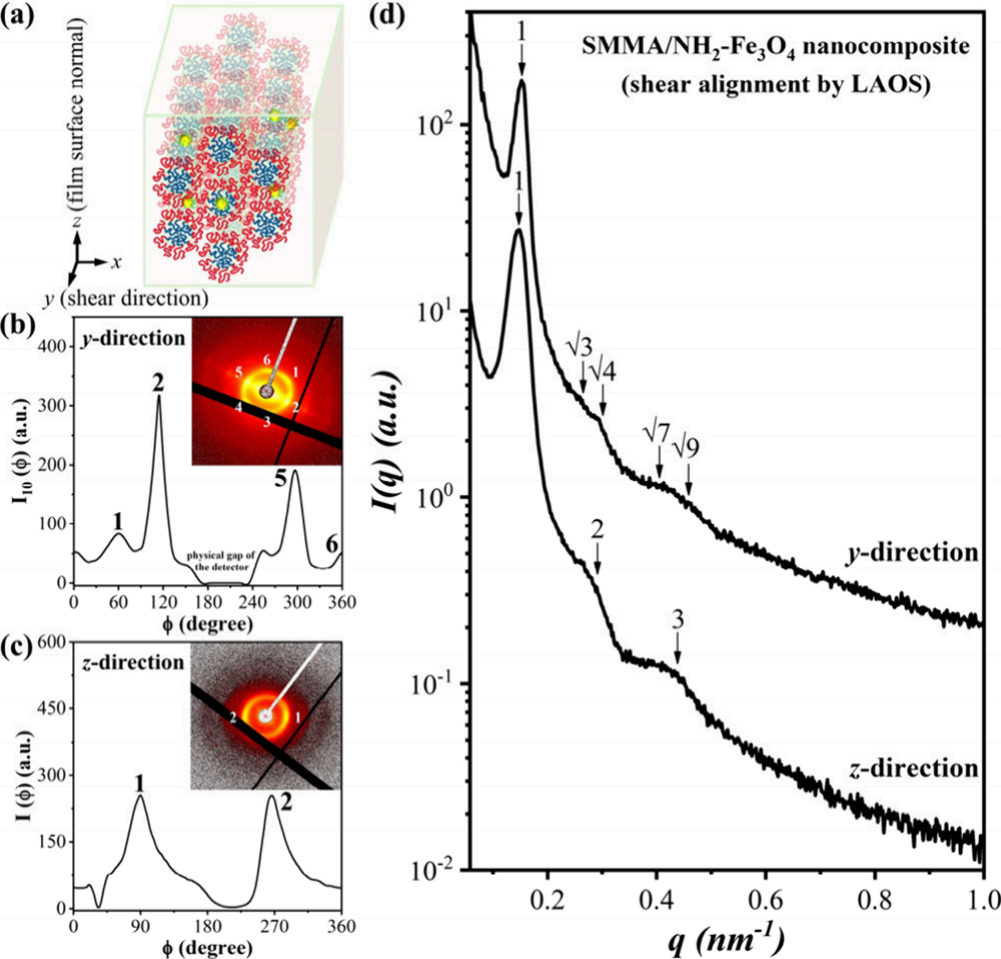
(a) Illustration of the shear-induced alignment in the HEX-forming SMMA/NH_2_–Fe_3_O_4_ nanocomposite film; (b-d) 2-D and 1-D SAXS patterns collected from the *y* and *z* directions and the corresponding azimuthal scan profiles.

Originally, we expected that subsequent magnetic manipulation of the hybridized nanoparticles would at least enhance the long-range order in the oriented HEX structure if no lattice symmetry transition occurred. Interestingly, magnetic treatment of the shear-aligned sample under a 350 mT magnetic field (applied along the *y* direction at 150 °C in an ultrahigh-purity nitrogen atmosphere for 12 h followed by cooling to 30 °C at a cooling rate of 5 °C/min) promoted the formation of a CR phase, as depicted in Figure 2a. The packing of cylindrical microdomains into a centered rectangular lattice with preferred orientation along the film surface normal was evidenced by the 2-D SAXS pattern viewed along the *z* direction (Figure 2b), showing four arcs in the quadrants (as well as four maxima located at the azimuthal angles of 65°, 110°, 250°, and 295°). According to a further identification of the CR phase by the corresponding 1-D SAXS profile displayed in Figure 2d, which showed (11), (13), and (15) reflection planes, the absence of (*hk*) peaks with *h* + *k* = 2*n* + 1 demonstrated the centered symmetry, as reported previously.^22^ On the other hand, the 2-D SAXS pattern viewed along the *y* direction (Figure 2c) displayed equatorial arcs originating from the vertical projection of the centered rectangular lattice. The corresponding 1-D SAXS profile (Figure 2d) exhibited reflections at integer-multiple *q* positions along the out-of-plane direction, which arose from the (00n) series of a vertically oriented centered rectangular lattice instead of from genuine lamellar stacking. An additional tilted-angle (∼5° tilt around the cylinder axis) SAXS measurement revealed noninteger peaks (Figure S2), further confirming that the observed structure corresponded to a vertically oriented CR phase. Moreover, the reproducibility of the observed symmetry transition was verified by SAXS measurements on independently prepared nanocomposite films subjected to identical LAOS and magnetic-field protocols (Figure S3).

**Figure 2:**
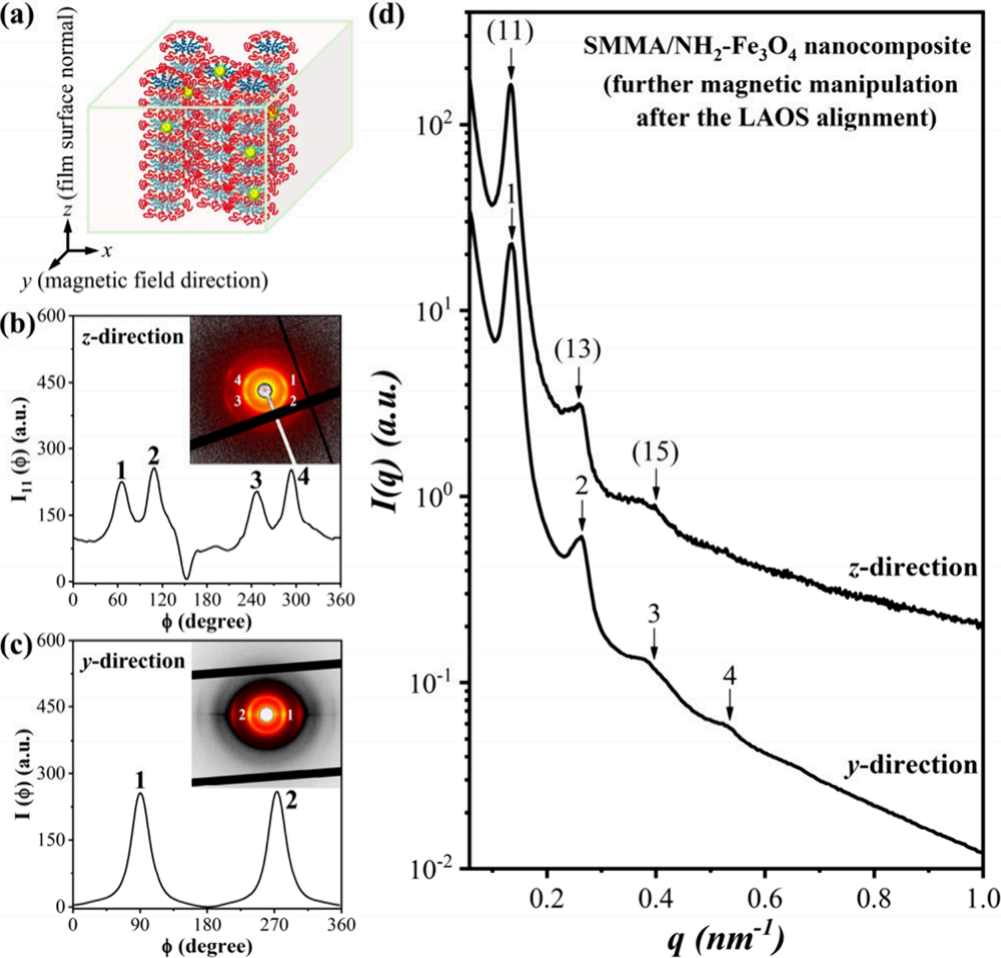
(a) Illustration of the magnetic-field-induced CR phase in the SMMA/NH_2_–Fe_3_O_4_ nanocomposite film; (b-d) 2-D and 1-D SAXS patterns collected from the *z* and *y* directions and the corresponding azimuthal scan profiles.

The persistence of the CR phase, rather than the recovery of the hexagonal packing, arose from the sequential action of shear alignment and magnetic-field application. The shear step first oriented the cylindrical microdomains parallel to the shear direction, imposing an in-plane anisotropy. Subsequent magnetic-field application amplified this anisotropy, elongating the microdomains along the field and yielding unequal lattice spacings (*a* ≠ *b*) that defined the CR symmetry. This rearrangement relieved local packing frustration and became energetically favored under coupled shear-magnetic field conditions. Furthermore, as demonstrated in Figure S4, the CR phase persisted after prolonged thermal annealing at 150 °C following removal of the magnetic field. Both samples, annealed for 3 days and 1 week, respectively, retained the characteristic CR reflections, although the latter exhibited a slightly decreased peak intensity. These results indicated that the CR phase represented a metastable yet long-lived state induced by the combined effects of shear-imposed orientation and magnetic-field-induced anisotropy. Complete recovery of the equilibrium HEX structure would require the elimination of both anisotropic contributions, which was not achieved under the present experimental conditions.

The SAXS analysis clearly revealed not only a magnetic-field-directed HEX-to-CR lattice symmetry transition (notably, this transition could not be induced without the preceding shear-aligned process and will be further discussed in Figure 5), but also a reorientation of the cylindrical microdomains, shifting from parallel to perpendicular alignment relative to the nanocomposite film surface. According to *q*_*hk*_ = 2π­[(*h*/*a*)^2^ + (*k*/*a*)^2^]^1/2^, the scattering peaks indexed for the CR phase were used to determine lattice parameters, yielding *a*, *b*, *l*, and γ (= 57.7, 81.6, and 50.0 nm, and 54.7°, respectively) where *l* = [(*a*/2)^2^ + (*b*/2)^2^]^1/2^ and γ = tan^–1^ (*b*/*a*)), as illustrated in Figure 3a. Meanwhile, the real-space observation of this CR phase as visualized by TEM is displayed in Figure 3b, through which the lattice parameters thus measured were consistent with those determined by SAXS.

**Figure 3:**
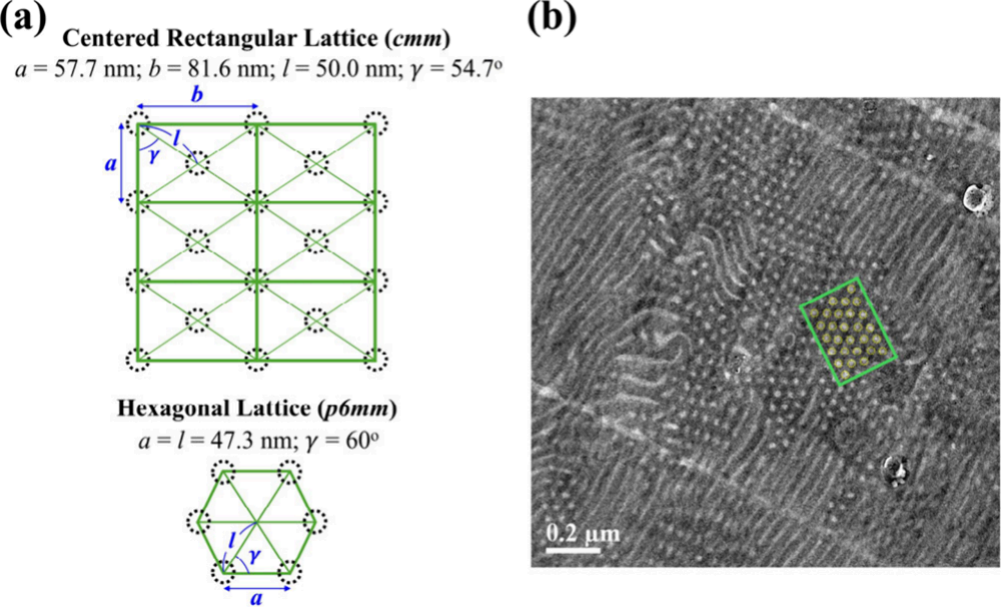
(a) Comparison of lattice parameters determined from the SAXS data shown in Figure 2, illustrating the transition from the original HEX phase to the magnetic-field-induced CR phase. (b) The corresponding TEM micrograph showing the CR phase, in which the light gray region corresponds to the PMMA phase, whereas the deep gray region represents the PS phase.

Our previous studies demonstrated that magnetic-field-directed assembly of magnetic nanoparticles within block copolymer-derived hexagonally packed cylinders triggers phase transitions, producing oriented lamellae and double gyroid phases.^30,31^ Here, we advance the applicability of this approach to controlled lattice-symmetry switching, achieving the transition from a hexagonal lattice to a centered rectangular lattice. Particularly, both the shear alignment prior to magnetic manipulation and the subsequent microdomain reorientation played significant roles in governing the lattice symmetry transition kinetics. It is noted that, to determine whether any intermediate structures appeared during magnetic field annealing, nanocomposite films were quenched after several annealing times (e.g., 3, 6, and 9 h) and characterized by SAXS (Figure S5). The sample annealed for 3 h still displayed a parallel-oriented HEX structure, whereas all subsequently annealed samples exhibited the vertically oriented CR phase. No coexistence or transient scattering features were observed throughout the annealing sequence, demonstrating that the transition proceeded through discrete orientational switching between parallel and perpendicular states. Such abrupt reorientation agreed with theoretical predictions and rheo-SAXS studies of cylinder-forming block copolymers under shear, which reported first-order-like orientational transitions without long-lived oblique states.^34–38^ For field-driven alignment, self-consistent-field calculations similarly predicted direct parallel → perpendicular reorientation in the absence of strong surface preference,^39,40^ consistent with our bulk-film conditions. A kinetic pathway is therefore proposed, as illustrated in Figure 4. Let us begin with the shear-aligned HEX structure, in which the NH_2_–Fe_3_O_4_-containing cylindrical PMMA microdomains were oriented along the *y* direction, as displayed in step (a). When a 350 mT uniform magnetic field was applied through the cylindrical axes at 150 °C, the magnetic field lines guided the NH_2_–Fe_3_O_4_ nanoparticles into an improved alignment within the cylindrical microdomains. In this case, magnetic interactions between magnetized nanoparticles located in adjacent PMMA cylinders reduced the intercylinder spacing along the *z* direction, as illustrated from step (b) to step (c). This process led to lattice symmetry breaking, transforming the hexagonal packing with higher symmetry into the lower-symmetry centered rectangular packing. However, as illustrated in panel (e), such a centered rectangular packing would result in nonuniform stretching of the PS block chains around each cylinder, incurring an entropic penalty from excessive chain stretching along the *x* direction and chain crowding at junction points in the *z* direction when filling the intercylinder voids. To alleviate chain crowding and thereby minimize excessive chain stretching of the PS block chains, microdomain reorientation occurred to induce significant chain relaxation, as shown in the transition from step (c) to step (d), because relying solely on local redistribution of block copolymer chains was likely ineffective in facilitating chain relaxation. Now, as illustrated in panel (f), substantial relaxation of the PS block chains was induced by microdomain reorientation, which consequently stabilized the CR phase. This stabilized CR phase remained preserved after the nanocomposite film cooled to 30 °C.

**Figure 4:**
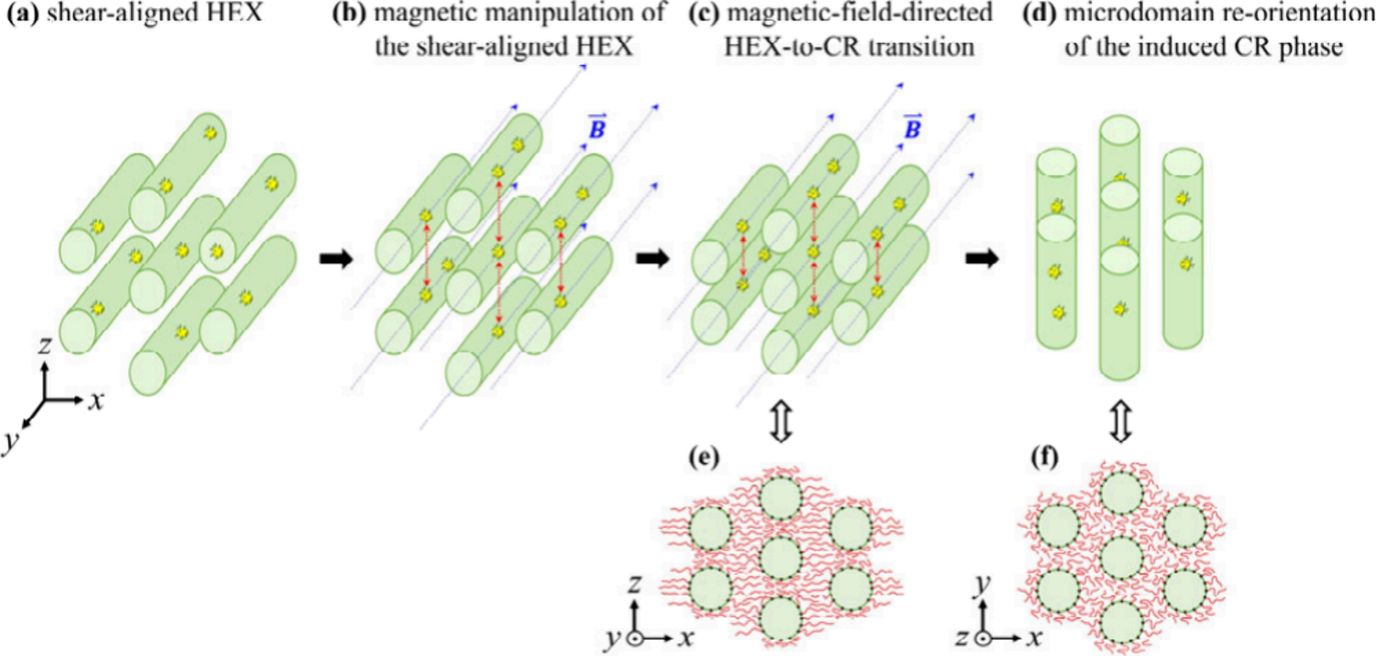
Schematic illustration of the kinetic pathway for the magnetic-field-directed HEX-to-CR lattice symmetry transition in the SMMA/NH_2_–Fe_3_O_4_ nanocomposite film.

It is worth noting that, in the absence of shear alignment, direct magnetic manipulation of the HEX-forming SMMA/NH_2_–Fe_3_O_4_ nanocomposite film with a 350 mT field led to an alternative kinetic pathway of the HEX-to-lamellae transition, as shown in Figure 5 (the reproducibility of this transition was verified in Figure S6). The appearance of paired arcs along the meridian in the 2-D SAXS pattern collected from the *y* direction (Figure 5b), together with the integral peak position ratio in the corresponding 1-D SAXS profile (Figure 5d), indicated the formation of an oriented lamellar structure with microdomains aligned parallel to the xy plane (i.e., the nanocomposite film surface). However, the 2-D SAXS pattern along the *z* direction (Figure 5c) displayed two anisotropic rings with the characteristic integral peak position ratio (Figure 5d), indicating that the lamellar microdomains were slightly tilted relative to the film normal such that interference from X-rays scattered by the alternating lamellae of PS and PMMA blocks could still be observed. The magnetic field imposed directional anisotropy through the alignment of NH_2_–Fe_3_O_4_ nanoparticles and their associated polymer interfaces, which decreased the interfacial free energy for lamellae oriented parallel to the field. This anisotropic alignment accounted for the exclusive formation of lamellae under magnetic-field application and was consistent with our previous findings,^30^ where the field-induced HEX-to-lamellae transition yielded a thermally stable lamellar morphology. Indeed, the thermodynamic stability of the lamellar structure was examined by temperature-dependent SAXS, which revealed reversible transition behavior during thermal cycling (Figure S7).

**Figure 5:**
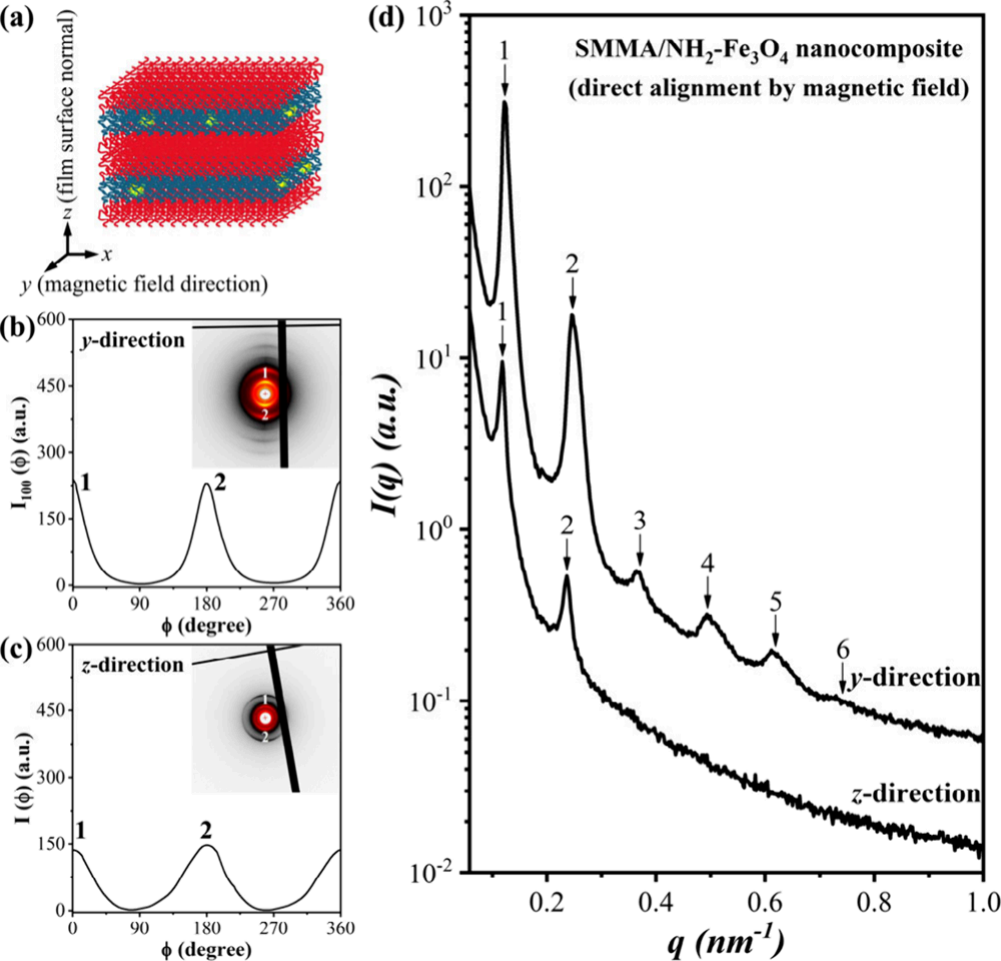
(a) Illustration of the magnetic-field-induced (without the preceding shear-aligned process) lamellae phase in the SMMA/NH_2_–Fe_3_O_4_ nanocomposite film; (b-d) 2-D and 1-D SAXS patterns collected from the *y* and *z* directions and the corresponding azimuthal scan profiles.

In summary, we introduced a strategy to control lattice symmetry in a PS-*b*-PMMA diblock copolymer hybridized with a low content of NH_2_–Fe_3_O_4_ magnetic nanoparticles by employing low-intensity magnetic fields as “non-contact tweezers”. When combined with LAOS prealignment, the magnetic field transformed the shear-aligned HEX lattice into an oriented CR phase, stabilized through microdomain reorientation that alleviated chain crowding and excessive stretching of the PS blocks. By contrast, field application to an unoriented HEX structure led to its reorganization into the thermodynamically stable lamellar structure. These results highlight that lattice symmetry and phase stability can be selectively tuned by coupling magnetic-field-induced anisotropy with the initial microdomain orientation, offering a versatile route for designing ordered nanostructures via combined shear and magnetic control.

## Supplementary Material



## Notes

Materials and nanocomposite film preparation; small-angle X-ray scattering measurement; transmission electron microscopy observation; large-amplitude oscillatory shear experiment; SAXS profiles of the neat SMMA and the SMMA/NH_2_–Fe_3_O_4_ nanocomposite film collected at 150 °C; tilted-angle SAXS profile of the CR-forming nanocomposite film collected along the *xy* plane; overlaid SAXS patterns for independently prepared CR-forming nanocomposite films; SAXS profiles of nanocomposite films thermally annealed at 150 °C for 3 days and 1 week after removal of the magnetic field; SAXS patterns of nanocomposite films quenched after several magnetic annealing times; overlaid SAXS patterns for independently prepared lamellae-forming nanocomposite films; temperature-dependent SAXS profiles of the lamellae-forming nanocomposite film collected in the absence of the magnetic field (PDF)
