# Supplementary material for: Low-Intensity Magnetic-Field-Directed Lattice Symmetry Transition to Induce the Centered Rectangular Cylinders in Diblock Copolymer/Magnetic Nanoparticle Nanocomposite Films
Source: ACS Macro Lett. 2025 Dec 16;15(1):40–5. doi: 10.1021/acsmacrolett.5c00594 (PMC12825363; doi:10.1021/acsmacrolett.5c00594)
Supplement: Supplementary file 1 [file mz5c00594_si_001.pdf]

Supporting Information

Low-Intensity Magnetic-Field-Directed Lattice Symmetry Transition to Induce the Centered Rectangular Cylinders in Diblock Copolymer/Magnetic Nanoparticle Nanocomposite Films

Wen-Hong Li,[1] Wen-Sheng Chiu,[1] Che-Yi Chu,[1]* Ying-Xuan Huang,[2] and Yeo-Wan Chiang[2]

[1]Department of Chemical Engineering, National Chung Hsing University, Taichung 402, Taiwan

[2]Department of Materials and Optoelectronic Science, National Sun Yat-sen University, Kaohsiung 804, Taiwan

*To whom correspondence should be addressed (C.-Y. Chu: cychu0123@dragon.nchu.edu.tw)

Materials and Nanocomposite Film Preparation.

The PS-*b*-PMMA diblock copolymer ($M_{n,PS}$ = 55,000, $M_{n,PMMA}$ = 22,000, and PDI = 1.08) studied here was purchased from Polymer Source, Inc. The volume fraction of the PMMA block (f_{PMMA}) was 25.9 vol%. The NH_2-Fe_3O_4 magnetic nanoparticles (with a mean diameter of 6.2 nm for the Fe_3O_4 core and a tethering density of 2.9 groups/nm^2 for the tethered NH_2 groups on each nanoparticle) prepared by glycine modification were purchased from Taiwan Advanced Nanotech, Inc.

For the preparation of the SMMA/NH_2-Fe_3O_4 nanocomposite film, the PS-*b*-PMMA diblock copolymer and the NH_2-Fe_3O_4 magnetic nanoparticles (= 0.38 vol% with respect to the selectively miscible PMMA phase) were dissolved in cosolvent of tetrahydrofuran and deionized water at 30 °C to obtain the thoroughly mixed solution. Subsequently, the solution was cast on a Petri dish under vacuum at 30 °C to yield the nanocomposite film with a controlled thickness of 1 mm.

Small-Angle X-ray Scattering (SAXS) Measurement.

The morphology of the SMMA/NH_2-Fe_3O_4 nanocomposite films was characterized by the small-angle X-ray scattering (SAXS) conducted at Endstation TLS23A1 and TPS25A1 of the National Synchrotron Radiation Research Center (NSRRC), Taiwan. At Endstation TLS23A1, the energy of the X-ray source and the sample-to-detector distance were 8 keV and 1.815 m, respectively. The scattering signals were collected by using a Pilatus-1MF detector of 981 x 1043 pixel resolution with a typical exposure time of 3 min. At Endstation TPS25A1, the energy of the X-ray source and the sample-to-detector distance were 15 keV and 3 m, respectively. An EIGER X 16M detector was used to collect the scattering signals with a typical exposure time of 30 s. The preferred microdomain orientations of the SMMA/NH_2-Fe_3O_4 nanocomposite films were studied by 2-D SAXS patterns. The corresponding

1-D SAXS profiles were output as a plot of the scattering intensity (I) versus the magnitude of the scattering vector, $q = (4\pi/\lambda)\sin(\theta/2)$ (θ = scattering angle). All 1-D SAXS profiles were corrected for the incident beam intensity, detector sensitivity, and background scattering.

Transmission Electron Microscopy (TEM) Observation.

The real-space morphology of the centered rectangular cylinders formed in the SMMA/NH_2-Fe_3O_4 nanocomposite film was visualized by using a JEOL JEM-2100 transmission electron microscopy (TEM) operated at 120 kV. The ultrathin section with a thickness of 50 nm was prepared by a microtome (where the cutting direction was parallel to the nanocomposite film surface) and then picked up onto carbon film supported copper grids. Subsequently, the ultrathin section was stained with the vapor of 0.5 wt% $RuO_{4(aq)}$ for 3 min for the observation of the NH_2-Fe_3O_4-containing cylindrical PMMA microdomains. In the micrograph, the PMMA phase appears as the light grey region and the PS phase corresponds to the deep grey region.

Large-Amplitude Oscillatory Shear (LAOS) Experiment.

To align the NH_2-Fe_3O_4-containing cylindrical PMMA microdomains of the SMMA/NH_2-Fe_3O_4 nanocomposite film, a Linkam CSS450 temperature-controlled shear system was used to prepare the oriented sample by large-amplitude oscillatory shear (LAOS). The sample was preshaped into a thin disk of suitable thickness and held in the gap between two glass windows. Subsequently, it was pressed between the two glass windows at 150 °C (well above the glass transition temperatures of the PS and PMMA blocks) and then sheared in an ultrahigh-purity nitrogen atmosphere for 1 h using an amplitude oscillatory step mode by the bottom window with the shear amplitude and frequency of 150 % and 0.1 Hz, respectively.

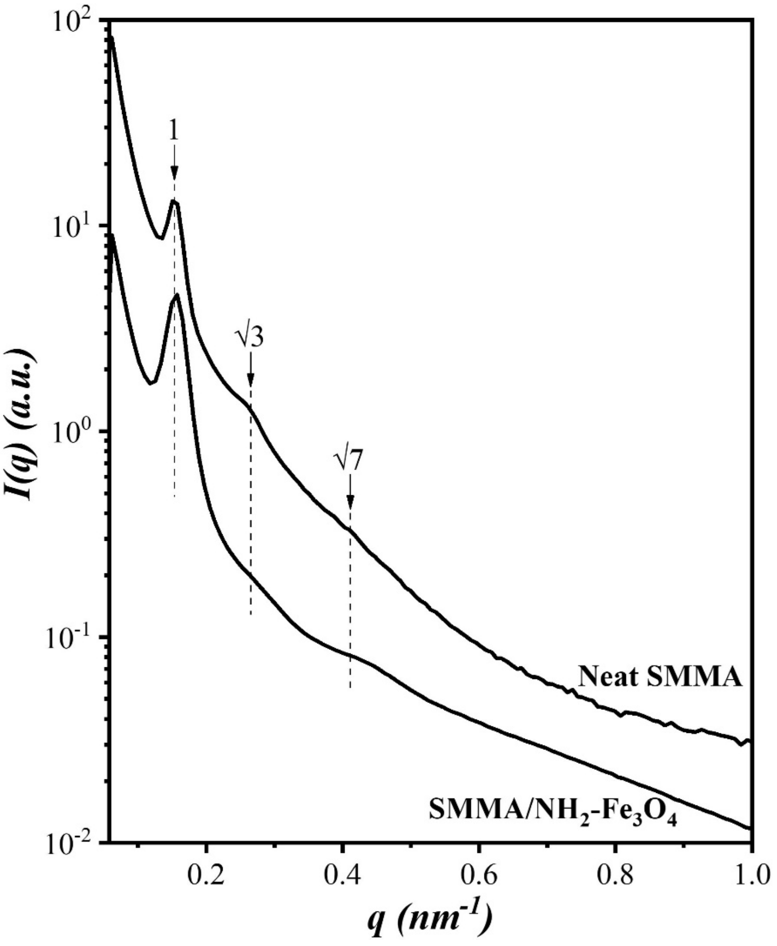

Figure S1. SAXS profiles of the neat SMMA diblock copolymer and the SMMA/NH_2-Fe_3O_4 nanocomposite film with 0.38 vol% NH_2-Fe_3O_4 magnetic nanoparticles collected at 150 °C. As identified by the SAXS profiles, a series of scattering peaks showing position ratios of 1: $3^{1/2}$: $7^{1/2}$ confirmed the formation of the HEX structure in both the samples. Overall, these SAXS results suggested good structural stability of the HEX morphology after incorporation of NH_2-Fe_3O_4 nanoparticles into the PMMA microdomains of the HEX-forming SMMA diblock copolymer.

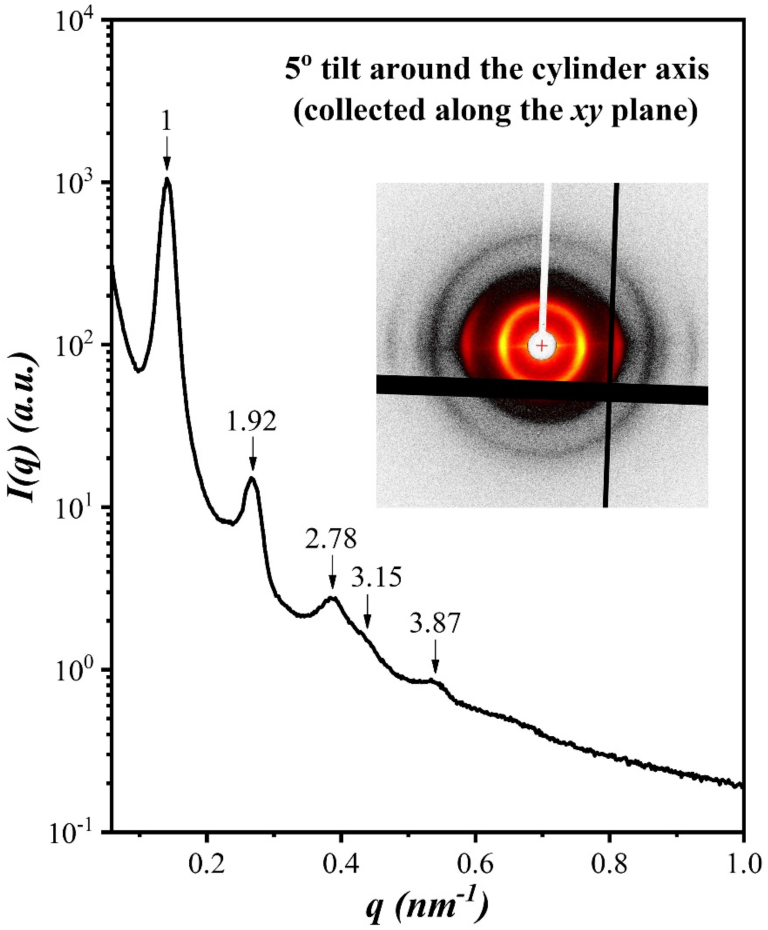

Figure S2. Tilted-angle (~ 5° tilt around the cylinder axis) SAXS profile of the CR-forming SMMA/NH_2-Fe_3O_4 nanocomposite film collected along the *xy* plane. The centered rectangular lattice was vertically oriented, with the cylinder axis parallel to the film normal (i.e., *z* direction, perpendicular to the *xy* plane). Non-integer reflections were observed, confirming that the integer-multiple peaks recorded along the *y* direction at normal incidence originated from the (00n) series of a vertically oriented centered rectangular lattice rather than from a lamellar morphology.

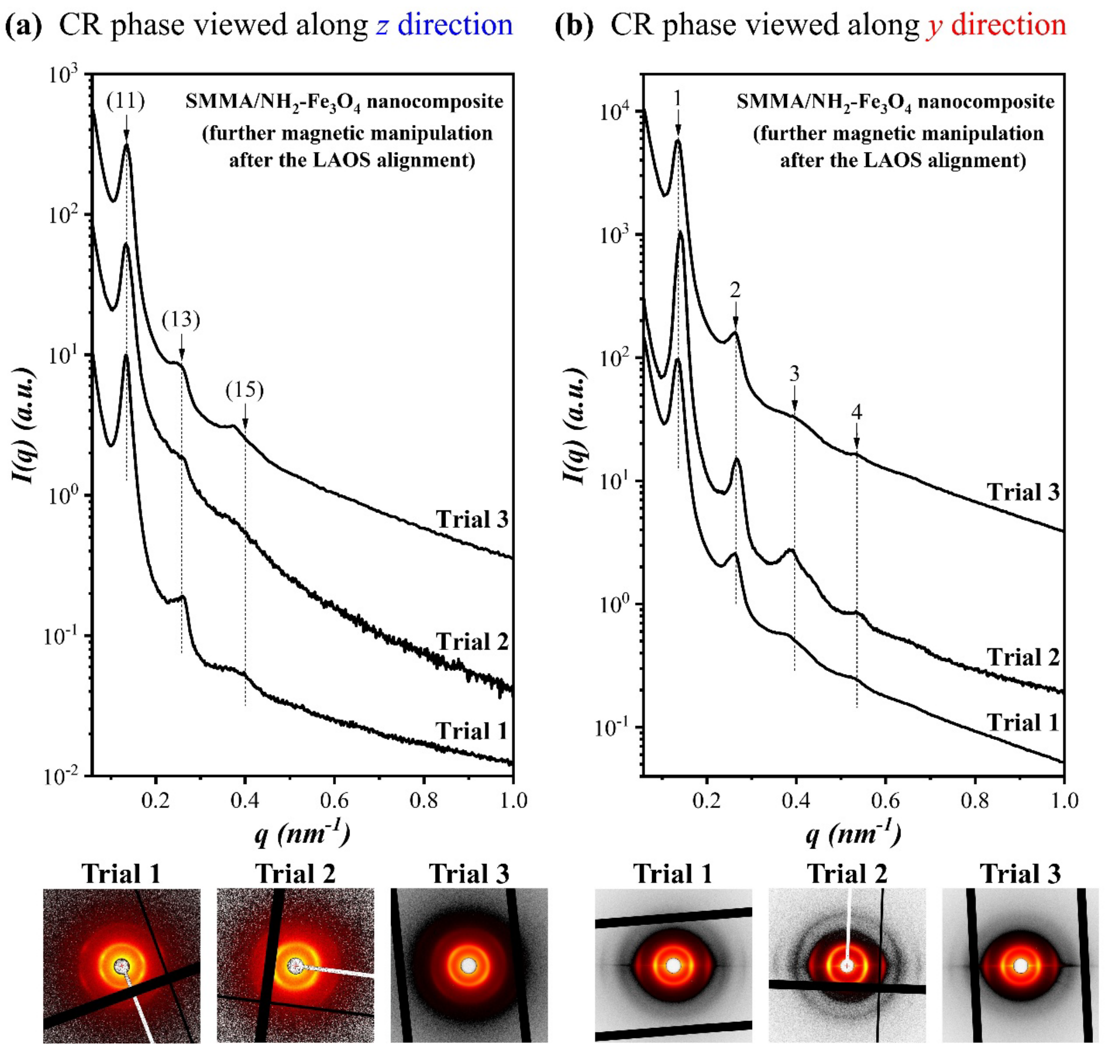

Figure S3. Overlaid 1-D and corresponding 2-D SAXS patterns viewed along the (a) *z* direction and (b) *y* direction for three independently prepared SMMA/NH_2-Fe_3O_4 nanocomposite films subjected to identical LAOS + magnetic-field protocols. The HEX-to-CR lattice symmetry transition occurred consistently, with reproducible peak positions and lattice parameters across samples, confirming that the symmetry change was intrinsic to the nanocomposite system rather than an isolated experimental artifact.

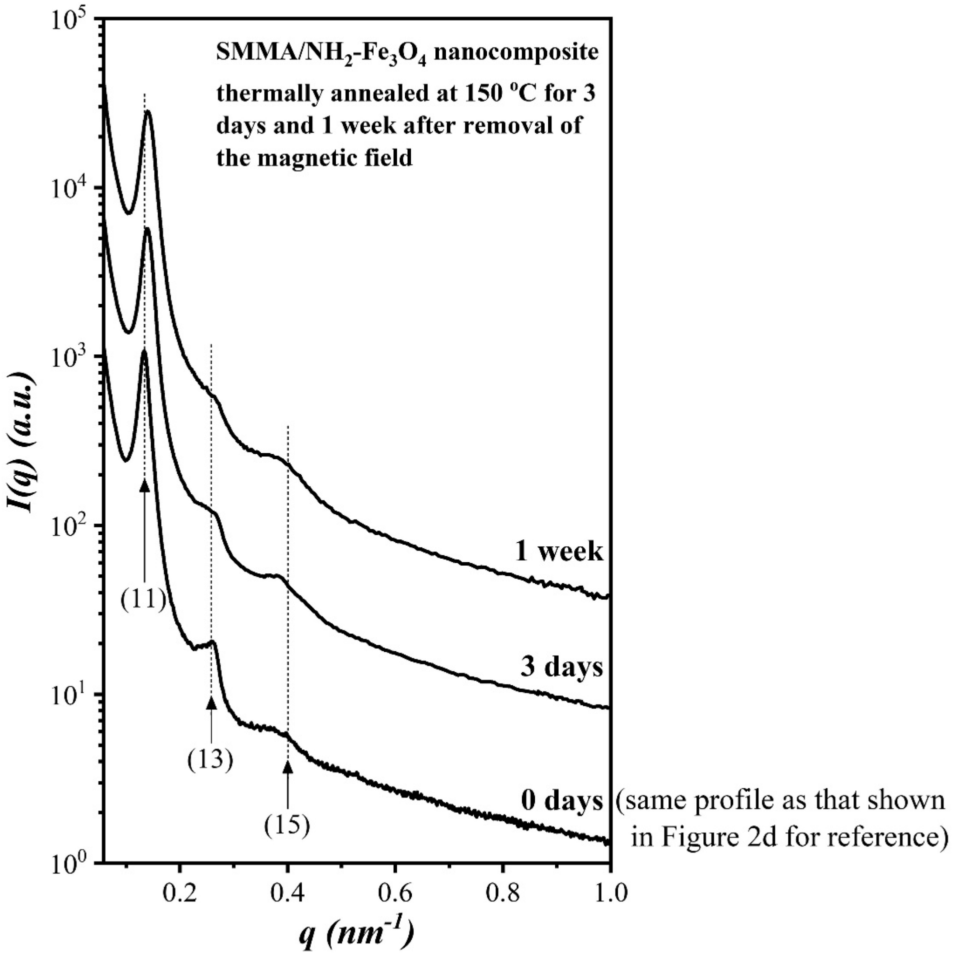

Figure S4. SAXS profiles of SMMA/NH$_2$-Fe$_3$O$_4$ nanocomposite films thermally annealed at 150 °C for 3 days and 1 week after removal of the magnetic field. Both samples retained the CR lattice reflections, with slightly decreased peak intensity after longer annealing, indicating a metastable yet long-lived state induced by the cooperative effects of shear-imposed orientation and magnetic-field-induced anisotropy.

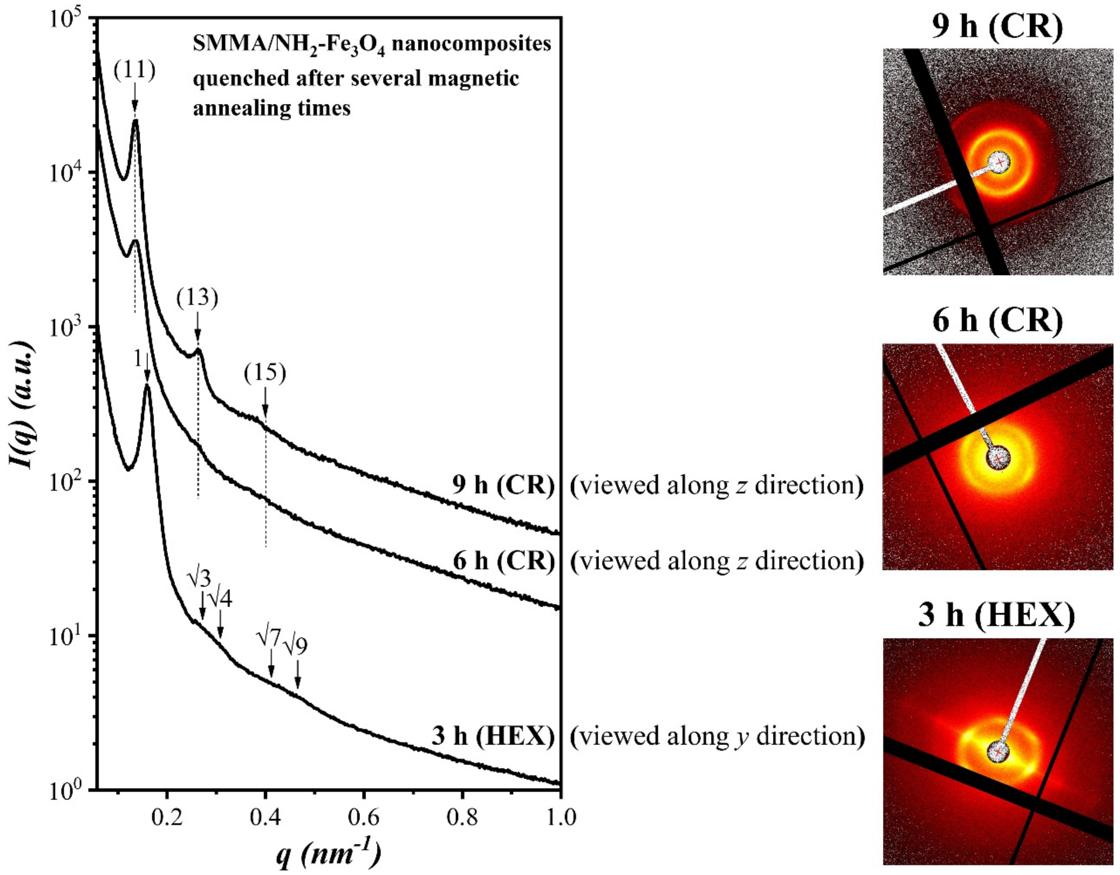

Figure S5. 1-D and corresponding 2-D SAXS patterns of SMMA/NH_2-Fe_3O_4 nanocomposite films quenched after several magnetic annealing times (e.g., 3, 6, and 9 h). The 3 h sample displayed a parallel-oriented HEX structure (viewed along the *y* direction), whereas all later samples exhibited the vertically oriented CR phase (viewed along the *z* direction). The absence of intermediate scattering features indicated a direct HEX→CR reorientation without a long-lived intermediate state.

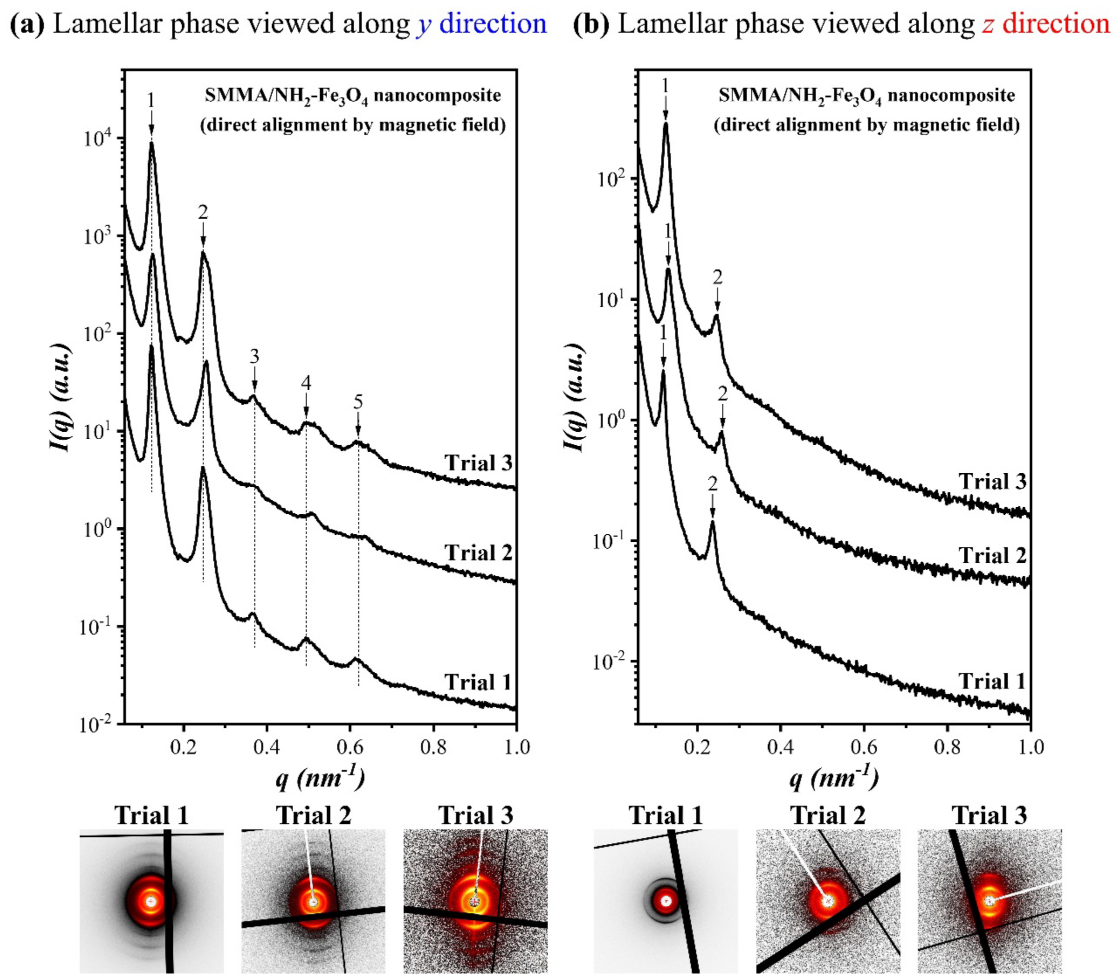

Figure S6. Overlaid 1-D and corresponding 2-D SAXS patterns viewed along the (a) *z* direction and (b) *y* direction for three independently prepared SMMA/NH_2-Fe_3O_4 nanocomposite films subjected to identical magnetic-field protocol, demonstrating reproducibility of the HEX-to-lamellae transition.

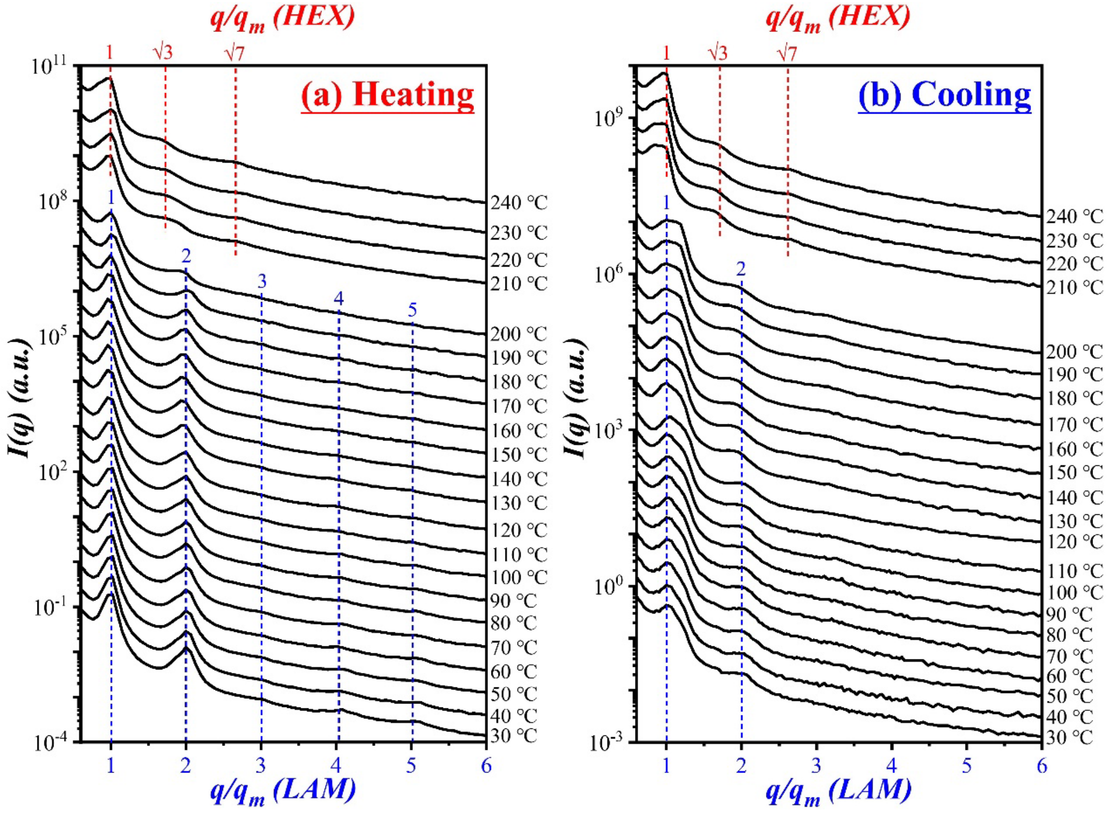

Figure S7. Temperature-dependent SAXS profiles of the lamellae-forming SMMA/NH_2-Fe_3O_4 nanocomposite film collected during thermal cycling in the absence of the magnetic field. Upon heating, the lamellar reflections persisted up to approximately 200 ºC and then transformed into the HEX structure between 210 and 240 ºC. During subsequent cooling, the HEX reflections remained from 240 to 210 ºC but reverted to lamellar reflections at around 200 ºC, which then persisted down to 30 ºC. These results demonstrated the reversible HEX ↔ lamellae transition and confirmed that the lamellar phase represented a thermodynamically stable morphology at lower temperatures.
